# Fruit bats adjust their foraging strategies to urban environments to diversify their diet

**DOI:** 10.1186/s12915-021-01060-x

**Published:** 2021-06-16

**Authors:** Katya Egert-Berg, Michal Handel, Aya Goldshtein, Ofri Eitan, Ivailo Borissov, Yossi Yovel

**Affiliations:** 1 Sagol School of Neuroscience, Tel Aviv University, 6997801 Tel Aviv, Israel; 2 School of Zoology, Faculty of Life Sciences, Tel Aviv University, 6997801 Tel Aviv, Israel; 3 Wissenschaftskolleg zu Berlin, Berlin, Germany

**Keywords:** Bats, Urbanization, GPS, Foraging, Behavior

## Abstract

**Background:**

Urbanization is one of the most influential processes on our globe, putting a great number of species under threat. Some species learn to cope with urbanization, and a few even benefit from it, but we are only starting to understand how they do so. In this study, we GPS tracked Egyptian fruit bats from urban and rural populations to compare their movement and foraging in urban and rural environments. Because fruit trees are distributed differently in these two environments, with a higher diversity in urban environments, we hypothesized that foraging strategies will differ too.

**Results:**

When foraging in urban environments, bats were much more exploratory than when foraging in rural environments, visiting more sites per hour and switching foraging sites more often on consecutive nights. By doing so, bats foraging in settlements diversified their diet in comparison to rural bats, as was also evident from their choice to often switch fruit species. Interestingly, the location of the roost did not dictate the foraging grounds, and we found that many bats choose to roost in the countryside but nightly commute to and forage in urban environments.

**Conclusions:**

Bats are unique among small mammals in their ability to move far rapidly. Our study is an excellent example of how animals adjust to environmental changes, and it shows how such mobile mammals might exploit the new urban fragmented environment that is taking over our landscape.

**Supplementary Information:**

The online version contains supplementary material available at 10.1186/s12915-021-01060-x.

## Background

Understanding the interactions between an animal and its environment and assessing how its behavior responds to changes in the environment is a major challenge in behavioral biology [1]. Movement is crucial for a large spectrum of behavioral processes and can serve as a measurable response to a combination of internal states and environmental changes such as shifts in resources, increase in conspecific competition, or changes in the habitat [2]. The analysis of movement is thus key for understanding how processes such as long-range navigation, orientation, and foraging strategies are affected by environmental changes, for example those induced by urbanization.

The ongoing massive growth of urban areas (i.e., urban sprawl) has resulted in the vanishing of vast natural habitats thus affecting many different species in various ways [3–7]. Although most animals are affected negatively, a minority of species can modify their behavior to the novel environment and adjust to a life in the city [8, 9]. Various behavioral adaptations have been reported in urban-dwelling animals. Studies conducted on birds, for example, show that individuals from urban populations were bolder and better at problem-solving than their rural counterparts [10–13]. Animals living in urban areas have also been reported to adjust their communication [14–19] and foraging [20–23]. Urban environments often offer new resources that differ in their distribution in comparison to the native environment of the animal. Urban foraging thus often requires strategy adjustments and specifically movement adjustments. Indeed, there is accumulating evidence that human activity and urbanization, in particular, affect animals’ movement and foraging patterns [23], but we are far from understanding the details of these effects.

Bats are among the most common mammals in cities [24, 25]. Previous studies on bats found that their response to urbanization is highly species-specific [24, 26–28]. Some species profit from urban habitats and human settlements, roosting in buildings, drinking from swimming pools [29–33], and reproducing more successfully [26], while the presence of other species dramatically declines in response to habitat loss and disturbance [21, 34–38]. There is little work on bats’ movement and foraging in urban environments. Geggie and Fenton suggested that *Eptesicus fuscus* bats in urban colonies spend more time out of their roosts (in comparison to rural conspecifics) and hypothesized that this might be a result of lower prey densities in the city, forcing these bats to fly farther [39]. Tomassini et al. suggested that changes in the cranial size of *Pipistrellus kuhlii* bats are a result of a diet shift due to anthropogenic activity [40].

Fruit bats (family Pteropodidae) are among the more prevalent families of bats in urban environments [41–45]. One hypothesized reason for this preference is its preferable micro-climate which is characterized in warmer temperatures suitable for these bats [46]. Cities have also been hypothesized to provide fruit bats a refuge from predation [47] and perhaps also to ease navigation due to the abundance of landmarks [44, 48]. Another suggested reason for fruit bat urban activity is fruit availability and diversity which is often richer and more stable year-round in cities in comparison to rural environments due to irrigation and planting [44]. Because the distribution of fruit trees and fruit species is much denser in urban environments than in rural or natural environments, we hypothesized that fruit bats will differ in their foraging strategy in these two environments. Specifically, we predicted that bats will fly less and visit trees near the roost. To test this, we documented and compared the foraging movement of Egyptian fruit bats (*Rousettus aegyptiacus*) in urban and rural environments.

The Egyptian fruit bat congregates in colonies of dozens to thousands of individuals and feeds on a wide range of fruit and nectar providing plants [49]. Many Egyptian fruit bats successfully exploit urban-roosting sites that can host large colonies (e.g., roofed parking lots) and can be seen foraging in gardens and backyards in Israel and the area [49, 50]. In parallel to their abundance in cities, many fruit bats still roost in rural environments. Fruit trees, the resource exploited by fruit bats, have very different distributions in urban and rural environments in Israel. Specifically, the diversity of fruit species per area is much larger in the city. This provides a fascinating opportunity to examine the differences in foraging in these two environments. We thus aimed to examine how Egyptian fruit bats that expertise on exploiting the city adjust their movement and foraging behavior. We used miniature onboard GPS devices to track the exact foraging behavior of fruit bats in urban and rural environments. We moreover reconstructed the bats’ diet by localizing and identifying the trees they ate from. This approach revealed that the environment (urban or rural) significantly affects foraging patterns. We further discovered that the location of the colony (urban or rural) does not determine the foraging grounds of its inhabitants, that is, many bats that do not roost in settlements nightly commute to forage within them. We suggest that fruit bats exploit cities in order to diversify their diets.

## Results

In total, we tracked 39 bats—19 from two rural colonies and 20 from two urban colonies for an average period of 8.1 ± 12.1 nights each. Because male and female bats can differ in their space exploitation [51], and because pregnant females might move differently, we narrowed our comparison to males only. Bats from urban colonies spent the great majority of their foraging time (72% on average) in urban areas, but interestingly, bats from rural colonies often foraged in urban environments, spending on average 45% of their time foraging in settlements. We used the Global Urbanization Footprint criterion (GUF, DLR 2016) [52–54] to distinguish between urban and rural foraging sites, and we quantified the percent of time each individual bat spent in each environment (see the “Methods” section).

### Exploratory urban foraging vs. fixed rural foraging

**Fig. 1:**
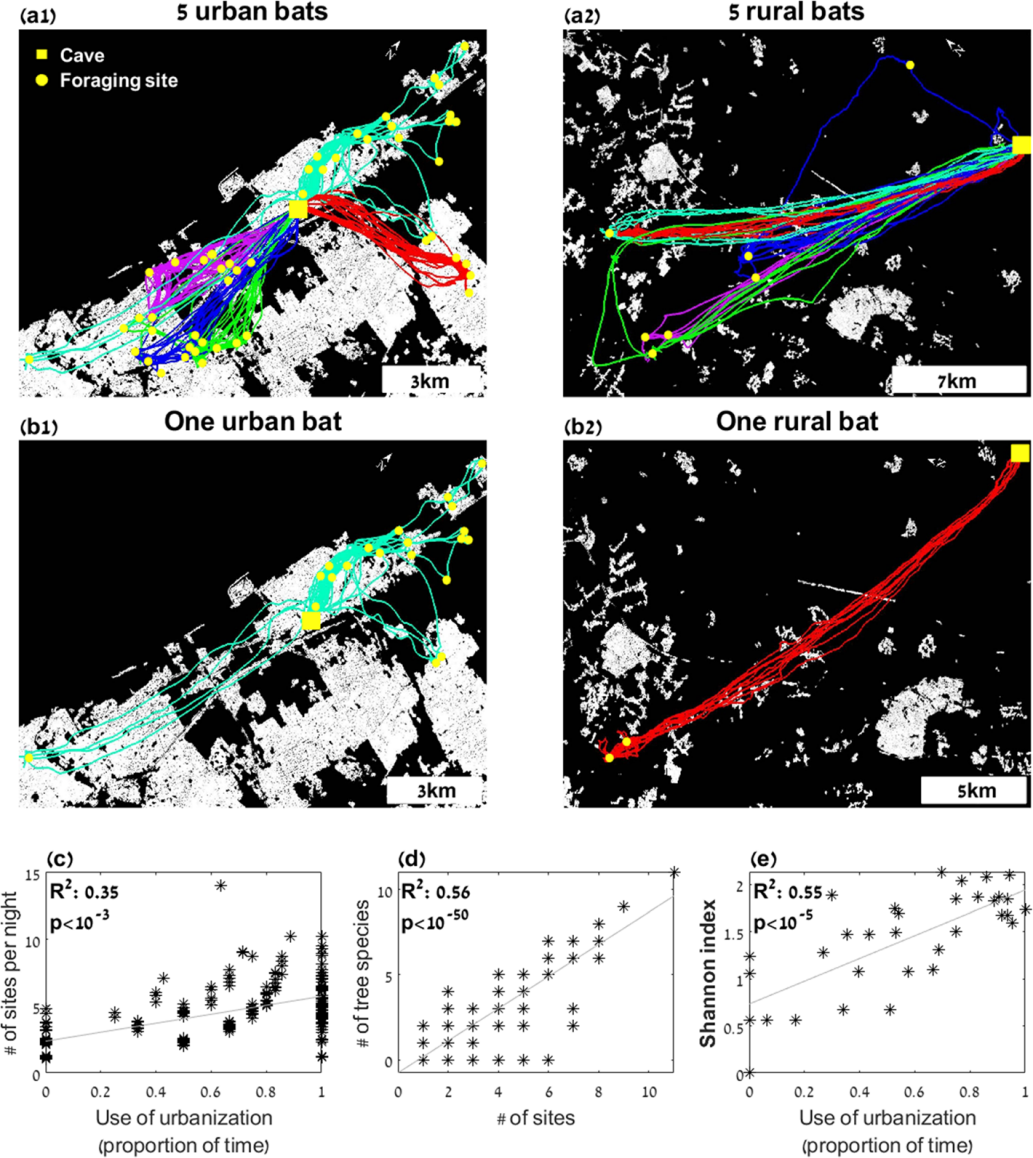
Roost and expertise shape foraging patterns. **a** The trajectories of 10 individuals: left—five bats from an urban colony who mostly forage in an urban area, and right—five bats from a rural colony who mostly forage in a rural area (each individual is colored differently). **b** The movement of one rural- and one urban-roosting bat. In both **a** and **b**, yellow dots depict foraging sites and yellow squares depict the roosts. **c** The number of sites visited by the bats as a function of the percent of time they spent in urban areas. In **c** and **d**, each point represents one night and all bats are overlaid. **d** The number of tree species visited as a function of the sites visited by the bat. **e** The Shannon index as a function of the percent of time they spent in urban areas. Each point represents a bat, black for bats from urban colonies and gray for bats from rural colonies. See main text for the statistical analysis of panels **c**–**e**

Independently of their roost, bats were much more diverse when foraging in urban environments, namely, they switched foraging sites more often, than in rural environments. Our results reveal a strong correlation between the amount of time a bat spends in urban environments and the number of foraging sites it visited. Bats visited up to three times more sites per night in urban environments (see Fig. 1a, b for bats’ trajectories and Fig. 1c; mixed effect GLM, P < 10^−3^, with the number of sites set as the explained variable; the percent of time spent in urban environments and the location of the roost—urban/rural—set as fixed effects; and with roost ID, bat ID, and season set as random effect intercepts). Using only bats that were tracked for at least 5 nights (ca. 54% of the data) did not alter the results (Additional File 1: Fig. S1), suggesting that our result is not an artifact of the tracking periods. Bats from urban colonies tended to switch sites more than bats from rural colonies (compare the gray and black points in Fig. 1c) but this difference was only nearly significant: P = 0.06 for the effects of roost location (urban or rural) and the interaction between roost location and the time spent in urbanization were not significant (P = 0.50). The result remained the same when examining the number of sites visited per hour (rather than over the entire night) to control for the need of rural bats to fly farther to reach the cities (P < 10^−3^, mixed effect GLM as above, but with the number of sites per hour set as the explained variable). Bats that foraged in urban environments routinely were also more prone to switch foraging sites on consecutive nights, while bats foraging in rural environments mostly returned to the same sites night after night (P = 0.02, mixed effect GLM as above, with the number of switches in consecutive nights set as the explained variable). The size of the settlement and the human population density where the bats foraged did not significantly affect the rate of site switching (P > 0.07, P > 0.12 when adding either the population size or density as fixed factors, to the above mixed effect GLM, with the number of visited sites set as the explained variable). This suggests that, in the research area, bats behave similarly in urban environments independently of their characteristics (e.g., large or small). The season of the year did not have a significant effect on the bats’ tendency to forage in urban or rural areas (P = 0.57, mixed effect GLM as above, with the percent of time spent in urban environments set as the explained variable, and the month of the year and the location of the roost—urban/rural—set as fixed factors. The interaction between season and roost location was also not significant P = 0.31).

We hypothesized that two main possible reasons can explain these environment-dependent differences in foraging: (1) increased competition in the urban environment drives bats to leave foraging sites more often; or (2) bats switch sites in urban environments to gain some benefit, such as to diversify their diet. We next examined both hypotheses.

#### Competition

Although fruit bats sometimes attempt to scrounge food from each other [55], these attempts seem to be part of a complex system of sociality [56], and our vast observations of foraging fruit bats did not reveal territorial behavior aiming to defend a tree and remove competitors. This did not surprise us, because trees offer much more fruit than visitors can consume on a given night. To validate this, we quantified the amount of ripe fruit on one of the common tree species eaten by the bats (*Ficus rubiginosa*, see the “Methods” section). Our assessment (based on n = 41 trees visited by our bats) suggests that on an average night, any of these trees in the region offers ~ 27 kg of ripe fruit. Note that this is the available fruit mass on a given night which is the important measurement for our purpose as it already takes consumption into account. That is, in the region of the study, every *Ficus rubiginosa* had on average ~ 27 kg of ripe fruit in every given night based on our assessments. This amount is enough to supply the nightly food demand of ~ 185 bats even if this was their *only* source of food. In our hundreds of observations, we have never observed more than ten individuals on a tree (the average number of bats was 1.9 ± 1.3, mean ± SD, n = 100 observations). The abundance of un-consumed fruit on the trees is also supported by the fact that a lot of fresh ripe fruit can be found on the ground under the trees regularly, suggesting that depletion does not drive the bats to switch foraging sites. Moreover, we identified ~ 200 fruiting (*Ficus rubiginosa*) trees in the area which should be able to feed more than 10,000 bats even if this was their only food source, while in reality, bats eat from dozens of other types of fruit (see Additional File 2: Table S1; because of *Ficus* seasonality, we assumed that ~ 25% of the trees have fruit at any moment). These are all rough estimates (see the “Methods” section), but in our calculations, we always tried to underestimate the number of trees and the amount of fruit, while we always overestimated the number of bats and their consumption, thus assuring that the conclusions are valid. We quantified the amount of fruit in one species that is easy to quantify (due to its relatively large fruit and spacious foliage), but it is important to note that settlements in the region are densely populated with fruit trees planted by the municipality or by private house-owners (Additional File 3: Fig. S2).

Moreover, an analysis of the bats’ social interactions at the foraging sites also suggests that competition was not driving them to switch foraging sites. Fruit bats often interact at the foraging sites and these interactions are accompanied by vocalizations (bats commonly land near a perching bat, a behavior that results in vocal communication [57]). We thus recorded audio continuously onboard nine bats (additional to the 39 above, the “Methods” section) to estimate the density of vocal interactions, as a proxy for bat density at foraging sites. Acoustic monitoring is a common method to assess bat density [58, 59], and we could estimate that ~ 90% of the social calls we recorded were emitted by conspecifics who were not interacting with the focal bat carrying the microphones. We could determine this because the intensity of a vocalization differs greatly when it is emitted by the individual carrying the microphone or by a remote conspecific. We could not determine whether one or more bats were calling, but it is unlikely that our result was driven by this. In light of our large sample size, it is unlikely that a local bias (e.g., one bat calling at one or a few locations) would generate the correlation that we observe (it could explain part of the noise that we see).

If competition drove the bats to switch foraging sites, we would expect a negative correlation between bat density and the time an individual bat spends at a foraging site. However, not only that this was not the case, we actually found a positive correlation between the abundance of social calls and the bats’ tendency to spend time on a foraging tree (P = 0.004, mixed effect GLM with the percent of time spent on the foraging tree set as the explained variable, the number of social calls set as a fixed factor, and the bat’s ID and date set as random effect intercepts). This finding is in line with the highly social nature of this species which causes them to seek conspecifics (bats are mostly alone on a tree). For example, when offering two food sources in captivity, most bats will aggregate at one source, consume it, and then move to the next one even though this is the less efficient strategy.

Moreover, there was no correlation between the bats’ mean propensity of tree switching and the mean bat density (Pearson correlation, R = 0.17, P > 0.6). While the GPS data showed that the bats switched trees more often in the first half of the night, the acoustic monitoring revealed that the number of bats on trees peaked at the middle of the night. Once again, this points against competition.

Using our onboard sensors, we also recorded continuous acceleration of these nine bats, which revealed that they spent the great majority of the time (> 80%) resting on the trees between occasional bouts of feeding (this is also the behavior we observe in the field). Once again, this contradicts competition, because if there was much competition over food, the bats would have been expected to be feeding or moving elsewhere but not resting most of the time. Noteworthy, bats from the same colony do not typically fly together to the same tree [60], so there is no group-defense incentive, as was suggested for other fruit-eating bats [61].

#### Dietary diversification

We hypothesized that the main benefit the bats could gain from changing their foraging strategy in urban environments is improving their diet, that is, acquiring more proteins and perhaps other nutrients that are essential for the species but often low in fruit [62]. We predicted that an attempt to improve the diet would be expressed by a higher diversity of fruit species in the diet of urban bats. We thus mapped the foraging sites visited by the bats in both rural and urban environments and identified the fruit they ate. Urban areas in warm countries like Israel are characterized by plentiful fruit trees, planted by both municipalities and individuals. We quantified the average number of fruit species in ten random urban squares of 0.5 × 0.5 km^2^ counting only species known to be consumed by fruit bats. Our estimates showed that such an area consists of a mean of 29.8 ± 4.6 bat-eatable fruit species, while in agricultural or wild rural environments, such an area will never contain more than a handful of fruiting tree species (often no more than one). The fact that in urban environments, the bats almost always switched fruit species when moving between sites strengthened the diet diversification hypothesis (Fig. 1d, notably, the Pearson correlation between sites visited and tree species visited was significant P > 10^−4^, R^2^ = 0.56. The abundance of tree types is far from uniform, so this correlation cannot be explained by random visitation). To quantify diet diversification, we compared diet diversity in urban and rural foraging environments using the Shannon and the Simpson diversity measurements. The diet diversity (measured by either parameter) was significantly correlated with the percent of time the bat spent foraging in urban environments (Fig. 1e, P = 0.0003 for the Shannon index, Additional File 4: Fig. S3, P = 0.0007 for the Simpson index; mixed effect GLM with the diversity set as the explained variable and the rest as above; results are reported for diversity estimates over 3 days, but they were significant for 4–5 days as well). In this analysis, there was also a significant correlation for the interaction term between the time spent in urban sites and the location of the colony, suggesting that bats that roost in the city might have some advantage in food diversification (P = 0.038 and P = 0.069 for the Shannon and Simpson respectively in the same GLM). Finally, if bats were driven by competition and did not actively try to diversify their diet, we would expect them to visit tree species according to their abundance, but this was clearly not the case. Comparing the distribution of available types of fruit in the area and the distribution of the trees actually visited reveals great differences between the two (Additional File 5: Fig. S4), suggesting that the bats are not simply hoping to the next available tree.

## Discussion

Understanding animal behavior in a rapidly changing world is one of the main goals of modern ecology. Specifically, it is crucial that we collect better information on how animals deal with fragmented urban environment, but in order to do so, we must acquire detailed information about the behavior of urban and rural populations, which is difficult to do, especially in small animals. Bats are of special interest due to their relative abundance in cities and their unique mobility among mammals. In this study, we GPS tracked the movement of fruit bats roosting and moving in urban and rural environments for the first time, allowing us to examine their precise foraging behavior. Our results demonstrate how fruit bats expertise in exploiting the city. When foraging in urban environments, bats exhibit a different foraging strategy switching foraging sites often, exhibiting much less stereotypical behavior than when foraging in rural environments.

We do not find evidence for competition or defense, and we hypothesize that switching foraging sites is a behavior adapted to the distribution of food in urban environments. The size or density of the settlement did not significantly affect the bats’ switching behavior. This is reasonable, because in Israel, the availability of different types of fruit within a short range is much higher in urban environments, including small villages, than in the countryside—where there is often plentiful fruit (e.g., in agricultural plantations), but where bats must fly far from one type of plantation to another. Indeed, we demonstrate that bats in urban environments switch fruit types very often, and by doing so, they achieve a more diverse diet. While in the country ca. 8 species of trees account for 70% of the bats’ diet, in the city, more than twice (ca. 17) species account for the same percentage (Additional File 6: Fig. S5). The city diet is characterized by many introduced species which are not common to rural areas such as *Ficus rubiginosa*. Bats turned to feeding in rural areas when a high-quality fruit such as *Diospyros kaki* was available in plantations. This was one of the only cases where bats from urban colonies exited cities to feed in rural areas. We did not directly measure how this behavior affects bats’ diet in terms of nutrients but as the bats seem to choose the species they visit, we hypothesize that food diversity is a proxy for diet quality. Switching foraging sites increases food diversity both directly, by switching fruit type, but probably also indirectly, by encountering new bats and potentially acquiring social information about additional resources. This idea of social information transfer was supported by our finding that bats seem to prefer trees with more conspecifics [55, 56]. Another possible advantage of switching foraging sites in urban environments is exploration of new sites (and repeated examination of familiar sites), which is probably more important in a rapidly changing environment such as a city (e.g., trees can be removed or pruned).

Although we cannot completely exclude some effect of competition over resources, this does not seem to be the main factor explaining urban exploration behavior, as trees typically have enough fruit to support many bats over many nights. Differences in predation risk, which have been used to explain animal behavior in urban environments (usually asserting that reduced predation in cities makes animals bolder) [19, 63, 64], are also not likely to be the reason for the behavior that we observe. Although we cannot overrule such differences completely, common species of owls, which are the main predators of fruit bats at the foraging site, can be found in both rural areas and settlements, but are probably more abundant in rural areas (e.g., *Bubo bubo* and *Tyto alba* [65]), and thus, we would expect bats foraging in rural areas to switch foraging sites more often.

Our results show that in a fragmented area, where human settlements are always available within a few kilometers, bats have to make two almost independent decisions: where to roost and where to forage. Of these two decisions, it is the bat’s foraging area and not its home that seems to determine how a bat behaves. Interestingly, we found that a substantial percent of the bats chose to live in rural colonies and commute to urban environments nightly to exploit them. Why these specific individuals do not roost inside urban environments is an open question. One possible hypothesis is the lack of stable roosts inside settlements. Urban fruit bats roost in roofed parking lots or abandoned buildings, but these roosts tend to be unstable due to human activity, and the bats are commonly driven out. An alternative explanation is that this choice reflects differences in behavioral types (often referred to as personality [66]) and that this might be another example of how personality shapes urban foraging behavior [67], where bolder individuals who are more susceptible to changes choose to roost inside settlements. Although urban-roosting bats occasionally forage in rural areas outside of the city, we observed very few urban-roosting bats that consistently foraged in the countryside. Even in the few cases that we observed, it seemed that this behavior was a result of the availability of highly attractive (ephemeral) fruit that cannot be found in cities—the bats flew to persimmon orchards.

## Conclusions

Among mammals, bats are unique in their immense movement capacity relative to their size. Indeed, we find that many fruit bats live outside settlements and commute nightly to exploit them. This is an example of how animals with high motility can live outside urban areas and still exploit them on demand. The better we understand how animals move and exploit urban environments, the better we will be able to draw conservation conclusions, which are essential in our rapidly changing world. Our study also demonstrates how animals can behave dramatically differently depending on the environment, exemplifying the importance of comparing animal behavior across backgrounds and contexts.

## Methods

### Animal model

The study was performed according to the permit or the Tel-Aviv University IACUC (Number: L-11-054). *Rousettus aegyptiacus* adult male bats were captured with permission from the Israeli National Parks Authority. Bats were collected from four colonies: two urban colonies (Herzelia cave (n = 18 bats) and our in-house university colony (n = 2 bats)) [55], both located deep inside the Tel-Aviv urban area, and two rural colonies (Beit Guvrin cave n = 17 and Segafim cave n = 2) both located in rural areas that are partially natural and partially agricultural. The two rural colonies are at least 10 km away from any city, but are surrounded by small agricultural settlements. Data were collected between January 2012 and February 2018. We monitored bats in all colonies along all seasons. For the movement comparison, we used data for 39 bats for which we had at least two nights of tracking (see full details in Table 1). Because we examined bats throughout the year and did not want to interfere with pregnant or lactating bats (which might also move differently), we only tracked males. For the audio and acceleration recordings to study competition, we used data from nine additional bats. We sampled bats from four colonies to avoid a strong colony bias, but it is important to note that all individuals were analyzed together as 39 independent individuals. Supporting the claim that these are independent individuals, we have shown in the past that the genetic relations of individuals in these fruit bat colonies are random (i.e., they are not more related than the average population [56]). We have also shown that fruit bats rarely follow each other in flight when emerging from the cave, strengthening their being independent foragers [60]. Moreover, these bats sometimes move to nearby roosts, further strengthening our claim that treating the individual colony as a sampling unit is meaningless. *The sample size used in this study is large in comparison to other studies that include tracking small animals.* We tracked the bats for a period of ~ 6.5 nights on average, which should be enough to detect environment-related differences. The fact that our data is spread over several years and over different seasons is an advantage, reducing the possibility of finding a difference in foraging that is a result of some transient difference between environments.

**Table 1:** Number of bats per analysis

Colony location	Bats with movement data (#)	Mean number of nights (#)	Total number of nights (#)	Mean ± SD time outside the roost (h)
Urban	20	6 6^a^	120 120^a^	6.0 ± 1.3
Rural	19	10.3 5.7^a^	196 109^a^	7.5 ± 0.9

^a^Number of nights for which we have tree analysis

### Animal tracking

Bats were caught in their roost using mist or hand nets. All bats were processed and tagged within 2 h and released at their cave. Tags were retrieved by collecting them on the ground after they fell off the animals. The tracking device was a GPS data-logger (Lucid Ltd., Israel, 30 × 20 × 4 mm). The device’s total weight (including battery, coating, and a telemetry unit—LB-2X 0.3 g, Holohil Systems Ltd. Carp, Ontario, Canada) was 11.8 g on average which accounted for 6.9% ± 0.42% of the weight of the bats (mean ± SD). The telemetry unit was attached to the device to assist in finding it once it fell of the bats. The devices were wrapped in polymorph for waterproofing and were attached to the bats using medical cement glue (Perma-Type Surgical Cement, AC103000, USA). After attaching, bats were held for about 5 min to allow the adhesive to dry and then placed in a cloth bag for another 15 min before release (see ref [68, 69] for full details). GPS positions were sampled at 10–15-s intervals.

To analyze bats’ behavior in response to conspecifics, we tracked additional nine bats from our open colony [70] for a period of four nights each using GPS devices that also include a microphone and an accelerometer (3 DOF) (Vesper Inc., with a Knowles microphone, FG series, A.S.D-tech). The tag’s average weight was 8.1 g accounting for 4.7% ± 0.29% of the bat’s body mass. GPS positions (sampled every 30 s) allowed us to extract the bats’ flight trajectories and the foraging trees that they visited (which were all visited by us). Continuous audio recordings were analyzed manually to detect all social calls emitted at the foraging site. Note that due to their low frequency [71], social calls can be picked up from a relatively long distance (at least 50 m) and thus all calls emitted (by any of the bats present on the foraging tree) were likely to be recorded. Because fruit bats often interact and vocalize when perching on foraging tress, we used the number of social calls as a proxy for the density of bats on the tree. Finally, acceleration analysis allowed us to distinguish between flight and perching bouts that were detected manually.

### Foraging and commute segmentation

Whenever we refer to commute in the study, we refer to the accumulated parts of the movement defined as commuting. Similar to our approach in a previous study [69], we used a combination of two indices, the straightness index [72] and the first passage time [73], to define foraging sites and to separate them from periods of commute.

- The straightness index (ranging between zero and one) is defined as the ratio between the minimal distance between two points and the length of the actual path traveled between these two points. Following our procedure in [69], the straightness index was calculated at each point along the trajectory with a window of 12 min. Values below 0.5 were defined as foraging (see [72] for details).
- The first passage time is defined as the total duration the animal spends within a given circle centered around any location along the trajectory. The first passage time was estimated for each location along the trajectory with a radius of interest of 50 m. The minimum first passage time for defining location as foraging site was set to 50 s (see [73] for details). These thresholds were motivated by the typical radius and time of flying around a foraging tree when also taking into account the GPS error. We have used it successfully in the previous study [69]. Any point along the trajectory that crossed one of the two thresholds (had a straightness index of less than 0.5 or a first passage time of more than 50 s) was defined as a moment of foraging. After identifying all potential foraging sites (i.e., connecting all locations in which foraging occurred), we omitted sites in which bats spent less than 30 s in total.

### Identification of foraging trees

The centers of the foraging sites were identified based on the bats’ GPS data by taking the mean over the x and y positions of all the locations that were defined as a foraging event.

We then visited and identified the trees in most of the bats’ foraging sites (72.5%). The tree closest to the center of the site was photographed and leaf samples were taken for consulting with experts. If there were more than one species of tree in close proximity, we could usually exclude non-relevant ones based on the season when the bat visited the site. Often, we could also find remains of chewed fruit or leaves under the trees (which are typically spat by fruit bats). In total, we mapped and identified 872 trees of 62 species (see Additional File 2: Table S1).

### Quantification of fruit on *Ficus rubiginosa*

We chose to quantify the amount of fruit on *Ficus rubiginosa* because it was one of the popular trees consumed by our bats, but not a tree that is extremely common and would thus make counting difficult. Forty-one *Ficus rubiginosa* trees visited by our bats were visited within 2 weeks of the bats’ visitation. A section of each tree was photographed and the number of ripe fruits (which can be detected according to their color (Additional File 7: Fig. S6)) was counted manually. The area of the section was estimated per photo using the size of a typical fruit as a scale. This provided us with the number of fruits per meter square which we extrapolated to the entire outer surface of the tree assuming it was a hemisphere (2∙pi∙R^2^). By including only the surface (and not the internal branches), we underestimate the amount of fruit. Moreover, we chose R = 4 m, even though most of our trees had radii of at least 6 m, once again to underestimate fruit quantity. The number of fruits per tree was translated to mass using the average fruit mass (1.9 g, measured on 500 fruits). To estimate the average amount of fruit offered nightly by all trees, we estimated that only a third of them offer ripe fruit at any given moment [74, 75]. The number of *Ficus rubiginosa* trees which we used for this estimate (200) is most likely a serious underestimate of the real number. This number was taken from the Tel-Aviv Municipality tree map which only maps ca. 50% of the trees and in the public domain only, and thus is, again, an underestimate (https://gisn.tel-aviv.gov.il/iView2js4/index.aspx?extent=3871338,3774019,3871706,3774169&layers=628,865&back=0&year=2019&opacity=0.8&filters=).

### Quantification of fruit species density in urban areas

To quantify the density of fruit species eaten by *Rousettus* in urban areas, we randomly chose 10 squares of 0.5 × 0.5 km^2^. We used the Tel-Aviv tree map (see above) and our own tree mapping to calculate how many fruit species (including only species known to be consumed by fruit bats) exist in each square. This is, again, an underestimation of the actual number of fruit species because of the map’s partiality (see above).

#### Urban vs. rural foraging sites

Urban foraging sites were defined as sites in urban regions, that is, sites that according to the GUF data are within a built-up area (a region featuring man-made building structures). The GUF data is a binary map (values of 255 for built-up areas and 0 for non-urban areas) generated from a global coverage of the Earth surface with TerraSARX/TanDEM-X radar data in 3 m ground resolution.

#### Use of urbanization

The time individual spent in urban environments was defined as the average time an individual spent in urban foraging sites in a single night or across all of its nights.

#### Behavioral variability over consecutive nights

Behavioral variability over consecutive nights was defined as the number of foraging site switches on consecutive nights. To this end, we estimated the number of changes required to transform one night’s array of foraging sites to the array of the consecutive night. The sets of foraging events on two consecutive nights were compared and a set with a minimal number of operations was sought to transform the first set into the second without maintaining the same order (i.e., the sets [1,2] and [2,1] are considered identical). The set of allowed operations includes insertion and deletion of sites, with both operations at a unit cost.

### Estimating diet diversity

Based on our monitoring of the fruit trees visited by the bats during their foraging, diversity indices were calculated to address the question of species diversity within the foraging sites of each community (city/country) and to compare the richness and evenness between them. Two of the most popular diversity indices are the Shannon index and the Simpson index, which use *P*_*i*_—the proportion of fruit species i [76]. We ran the same analysis on windows of 3–5 days and got a significant correlation with urbanity in all cases.

Shannon index:

$$ H=-\sum {P}_i\ln \left({P}_i\right) $$

Simpson index:

$$ D=-\frac{1}{\sum {P_i}^2} $$

### Statistics

Generalized linear mixed effect models were used (Matlab 2018) to assess the effect of different parameters on foraging and movement. The specific factors used in each analysis are described in the main text. All random effects were random intercepts. Count variables (e.g., the number of visits or tree species per night) were modeled using a Poisson distribution. To correct for possible seasonal biases, we added the month as an additional random effect in all statistical analyses.

## Supplementary Information

**Additional File 1: Figure S1**. Urban bats visit more sites per night. The number of sites visited by bats that were tracked for at least 5 nights as a function of the percent of time they spent in urban areas. Each point represents one night. Each point represents a bat, black for bats from urban colonies and grey for bats from rural 241 colonies.

**Additional File 2: Table S1**. Fruit species visited by bats in our study.

**Additional File 3: Figure S2**. Fruit trees available in urban environments. We color-coded all trees around the Herzelia cave where most of our urban bats came from (vegetation that was not color-coded is mostly comprised of fields). The great majority of these trees offer fruit that is consumed by fruit bats. Trees were identified using a green-color filter while validating our classification several patches with high-resolution images. We attempted to underestimate the identified trees in the image.

**Additional File 4: Figure S3**. Urban bats diversify their diets. The Simpsons index as a function of the percent of time they spent in urban areas. Each point represents a bat.

**Additional File 5: Figure S4**. Urban bats select the tree-types they visit. The graph shows the distribution of fruit trees in the city (blue line) with the trees ordered from the most to the least common species (left-right); while in red we present the actual visitation rate for each species. It is very clear that the visitation does not follow the distribution (we highlight a few of the most popular species).

**Additional File 6: Figure S5**. Urban bats visit a larger variety of fruit types. The accumulated percentage of the feeding according to the number of fruit species in urban and rural bats (based on Additional file 1: Table S1).

**Additional File 7: Figure S6**. Fruit bats select ripe fruit. A fruit bat feeding on a *Ficus rubiginosa* tree. Only red fruit (like the one in the bat’s mouth) was marked as ripe when estimating the amount of fruit.

## Data Availability

The datasets generated and/or analyzed during the current study are available in the Mendeley repository, 10.17632/brrpng6gzy.1 [77].
