# Supplementary material for: Fruit bats adjust their foraging strategies to urban environments to diversify their diet
Source: BMC Biol. 2021 Jun 16;19:123. doi: 10.1186/s12915-021-01060-x (PMC8210355; doi:10.1186/s12915-021-01060-x)
Supplement: Supplementary file 1 — Additional File 1: Figure S1. Urban bats visit more sites per night. The number of sites visited by bats that were tracked for at least 5 nights as a function of the percent of time they spent in urban areas. Each point represents one night. Each point represents a bat, black for bats from urban colonies and grey for bats from rural 241 colonies. [file 12915_2021_1060_MOESM1_ESM.docx]

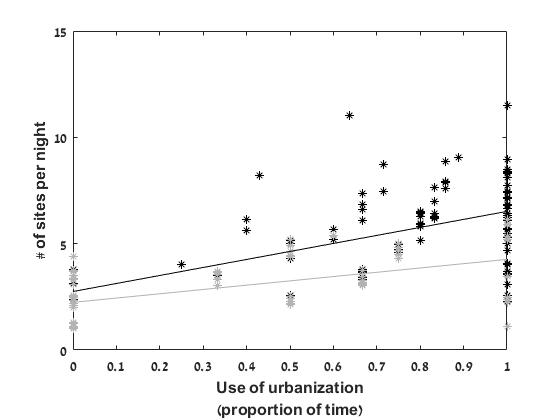
 **Fig. 1.** **Urban bats visit more sites per night.** The number of sites visited by bats that were tracked for at least 5 nights as a function of the percent of time they spent in urban areas. Each point represents one night. Each point represents a bat, black for bats from urban colonies and grey for bats from rural 241 colonies.
