# Supplementary material for: Fruit bats adjust their foraging strategies to urban environments to diversify their diet
Source: BMC Biol. 2021 Jun 16;19:123. doi: 10.1186/s12915-021-01060-x (PMC8210355; doi:10.1186/s12915-021-01060-x)
Supplement: Supplementary file 2 — Additional File 2: Table S1. Fruit species visited by bats in our study. [file 12915_2021_1060_MOESM2_ESM.docx]

**Table. 1. Fruit species visited by bats in our study**

|  | **Tree species** | ***% of visits in the city*** | ***% of visits in the country*** |
| --- | --- | --- | --- |
| **1** | *Diospyros kaki* | 0.65 | 23.93 |
| **2** | *Eucalyptus camaldulensis* | 2.12 | 13.51 |
| **3** | *Ficus microcarpa* | 9.13 | 8.11 |
| **4** | *Malus domestica* | 0.65 | 5.80 |
| **5** | *Punica granatum* | 1.80 | 5.02 |
| **6** | *Ficus religiosa* | 5.06 | 4.63 |
| **7** | *Ziziphus spina-christi* | 0 | 4.63 |
| **8** | *Citrus X sinensis* | 0.49 | 4.24 |
| **9** | *Ceratonia siliqua* | 0.33 | 3.47 |
| **10** | *Dalbergia sissoo* | 0.49 | 2.32 |
| **11** | *Melia azedarach* | 6.20 | 1.93 |
| **12** | *Phoenix dactylifera* | 5.71 | 1.93 |
| **13** | *Ficus sycomorus* | 2.44 | 1.93 |
| **14** | *Ficus carica* | 0.33 | 1.93 |
| **15** | *Prunus armeniaca* | 0.16 | 1.93 |
| **16** | *Celtis sinensis* | 1.79 | 1.54 |
| **17** | *Populus euphratica* | 0.16 | 1.54 |
| **18** | *Persea americana* | 0 | 1.16 |
| **19** | *Prunus amygdalus n.p.a* | 0 | 1.16 |
| **20** | *Ficus benghalensis* | 2.77 | 0.77 |
| **21** | *Trachycarpus fortunei* | 1.30 | 0.77 |
| **22** | [*Morus*](https://en.wikipedia.org/wiki/Morus_nigra) *nigra* | 1.14 | 0.77 |
| **23** | *Tamarix aphylla* | 1.14 | 0.77 |
| **24** | *Tipuana tipu* | 1.14 | 0.77 |
| **25** | *Ficus lyrata* | 0.65 | 0.77 |
| **26** | *Carya illinoinensis* | 0.49 | 0.77 |
| **27** | *Prunus persica* | 0.49 | 0.77 |
| **28** | *Vitis vinifera* | 0 | 0.77 |
| **29** | *Ficus neriifolia* | 7.01 | 0.38 |
| **30** | *Syagrus romanzoffiana* | 5.54 | 0.38 |
| **31** | *Washingtonia robusta* | 2.77 | 0.38 |
| **32** | *Schinus molle* | 0.81 | 0.38 |
| **33** | *Cercis siliquastrum* | 0.33 | 0.38 |
| **34** | *Muntingia calabura* | 0.16 | 0.38 |
| **35** | *Ficus rubiginosa* | 5.87 | 0 |
| **36** | *Washingtonia filifera* | 4.40 | 0 |
| **37** | *Eriobotrya japonica* | 2.28 | 0 |
| **38** | *Ficus elastica* | 2.12 | 0 |
| **39** | *Cycas revoluta* | 2.12 | 0 |
| **40** | *Dypsis Decaryi* | 1.96 | 0 |
| **41** | *Mangifera indica* | 1.63 | 0 |
| **42** | *Sabal palmetto* | 1.63 | 0 |
| **43** | *Phoenix canariensis* | 1.30 | 0 |
| **44** | *Albizia lebbeck* | 1.30 | 0 |
| **45** | *Ficus benjamina* | 1.30 | 0 |
| **46** | *Dimocarpus longan* | 1.30 | 0 |
| **47** | *Citharexylum spinosum* | 1.14 | 0 |
| **48** | *Litchi chinensis* | 0.98 | 0 |
| **49** | *Bombax ceiba* | 0.82 | 0 |
| **50** | *Brachychiton acerifolius* | 0.81 | 0 |
| **51** | *Schefflera actinophylla* | 0.81 | 0 |
| **52** | *Jacaranda mimosifolia* | 0.81 | 0 |
| **53** | *Psidium cattleyanum* | 0.65 | 0 |
| **54** | *Carica papaya* | 0.65 | 0 |
| **55** | *Pyrus* | 0.65 | 0 |
| **56** | *Hovenia dulcis* | 0.49 | 0 |
| **57** | *Alectryon tomentosus* | 0.49 | 0 |
| **58** | *Callistemon phoeniceus* | 0.33 | 0 |
| **59** | *Averrhoa carambola* | 0.33 | 0 |
| **60** | *Pistacia atlantica* | 0.16 | 0 |
| **61** | *Diospyros nigra* | 0.16 | 0 |
| **62** | *Physalis peruviana* | 0.16 | 0 |
