# Supplementary material for: Fruit bats adjust their foraging strategies to urban environments to diversify their diet
Source: BMC Biol. 2021 Jun 16;19:123. doi: 10.1186/s12915-021-01060-x (PMC8210355; doi:10.1186/s12915-021-01060-x)
Supplement: Supplementary file 3 — Additional File 3: Figure S2. Fruit trees available in urban environments. We color-coded all trees around the Herzelia cave where most of our urban bats came from (vegetation that was not color-coded is mostly comprised of fields). The great majority of these trees offer fruit that is consumed by fruit bats. Trees were identified using a green-color filter while validating our classification several patches with high-resolution images. We attempted to underestimate the identified trees in the image. [file 12915_2021_1060_MOESM3_ESM.docx]

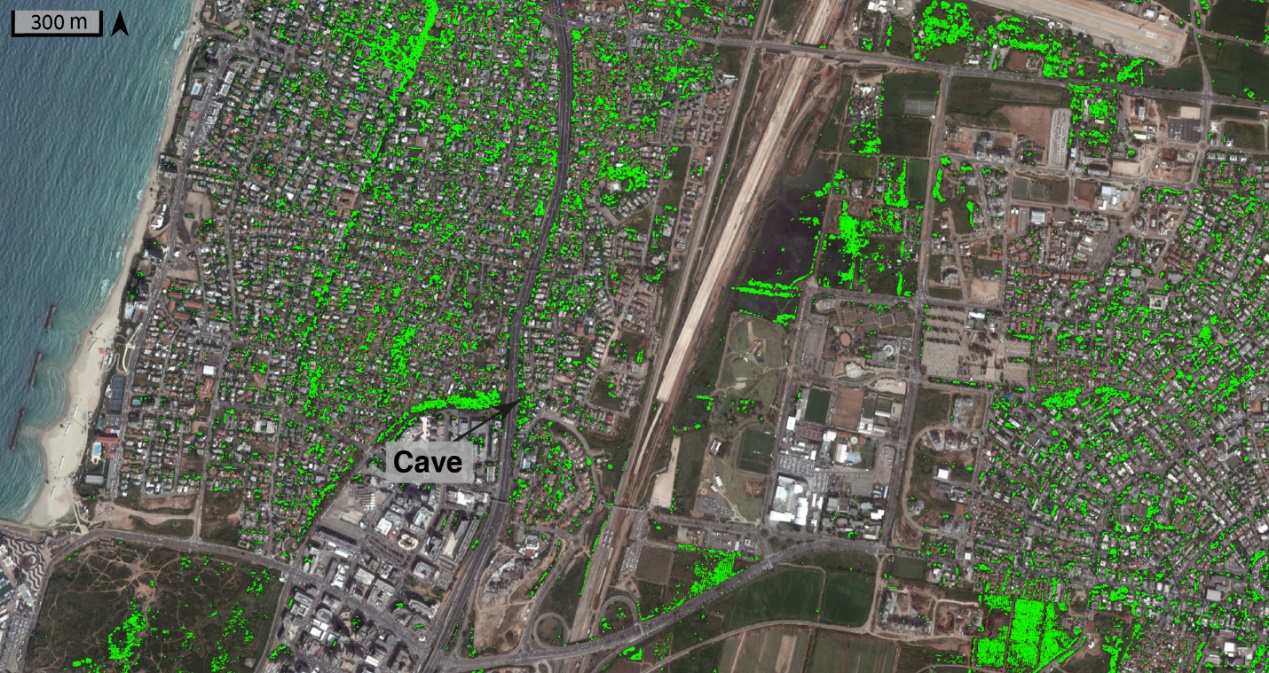


**Fig. 2.** **Fruit trees available in urban environments.** We color-coded all trees around the Herzelia cave where most of our urban bats came from (vegetation that was not color-coded is mostly comprised of fields). The great majority of these trees offer fruit that is consumed by fruit bats. Trees were identified using a green-color filter while validating our classification several patches with high-resolution images. We attempted to underestimate the identified trees in the image.
