# Supplementary material for: Fruit bats adjust their foraging strategies to urban environments to diversify their diet
Source: BMC Biol. 2021 Jun 16;19:123. doi: 10.1186/s12915-021-01060-x (PMC8210355; doi:10.1186/s12915-021-01060-x)
Supplement: Supplementary file 4 — Additional File 4: Figure S3. Urban bats diversify their diets. The Simpsons index as a function of the percent of time they spent in urban areas. Each point represents a bat. [file 12915_2021_1060_MOESM4_ESM.docx]

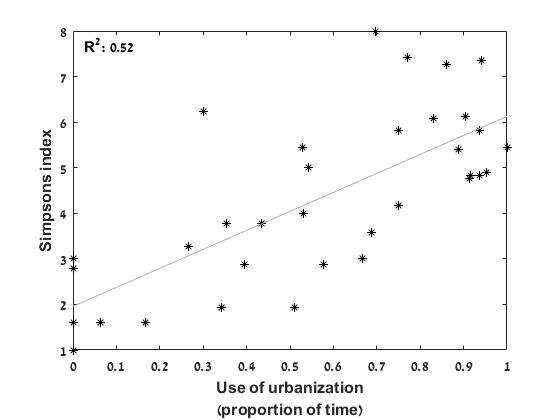


**Fig. 3.** **Urban bats diversify their diets.** The Simpsons index as a function of the percent of time they spent in urban areas. Each point represents a bat.
