# Supplementary material for: Fruit bats adjust their foraging strategies to urban environments to diversify their diet
Source: BMC Biol. 2021 Jun 16;19:123. doi: 10.1186/s12915-021-01060-x (PMC8210355; doi:10.1186/s12915-021-01060-x)
Supplement: Supplementary file 5 — Additional File 5: Figure S4. Urban bats select the tree-types they visit. The graph shows the distribution of fruit trees in the city (blue line) with the trees ordered from the most to the least common species (left-right); while in red we present the actual visitation rate for each species. It is very clear that the visitation does not follow the distribution (we highlight a few of the most popular species). [file 12915_2021_1060_MOESM5_ESM.docx]

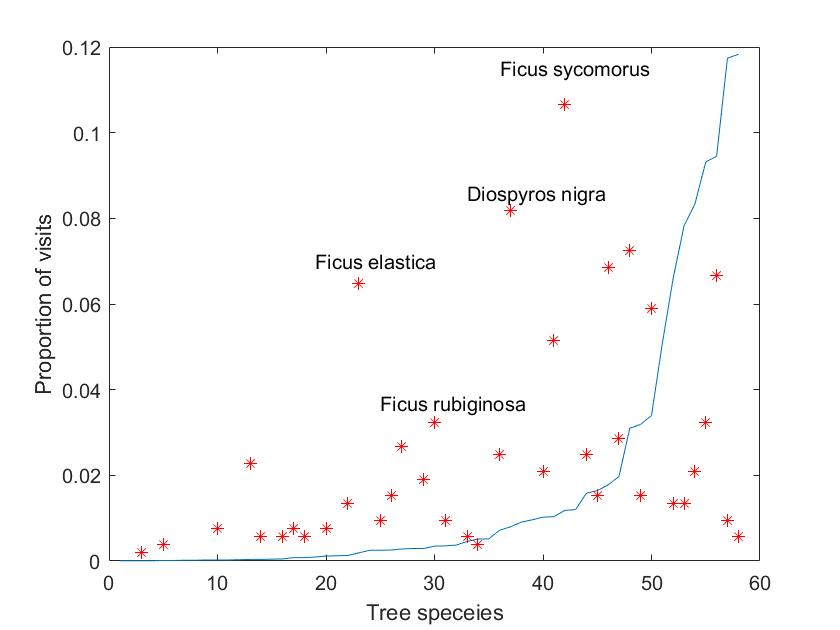


**Fig. 4.** **Urban bats select the tree-types they visit.** The graph shows the distribution of fruit trees in the city (blue line) with the trees ordered from the most to the least common species (left-right); while in red we present the actual visitation rate for each species. It is very clear that the visitation does not follow the distribution (we highlight a few of the most popular species).
