# Supplementary material for: Fruit bats adjust their foraging strategies to urban environments to diversify their diet
Source: BMC Biol. 2021 Jun 16;19:123. doi: 10.1186/s12915-021-01060-x (PMC8210355; doi:10.1186/s12915-021-01060-x)
Supplement: Supplementary file 6 — Additional File 6: Figure S5. Urban bats visit a larger variety of fruit types. The accumulated percentage of the feeding according to the number of fruit species in urban and rural bats (based on Additional file 1: Table S1). [file 12915_2021_1060_MOESM6_ESM.docx]

**
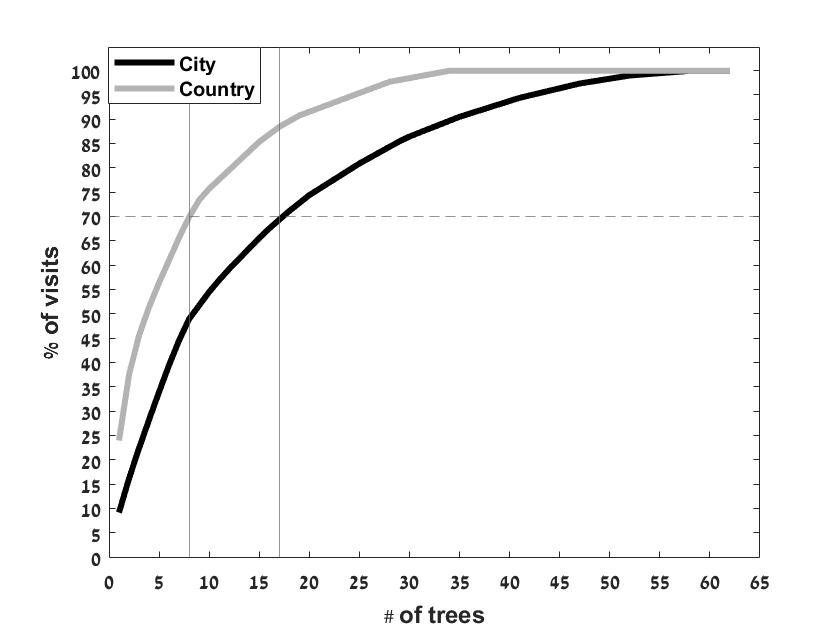
**

**Fig. 5. Urban bats visit a larger variety of fruit types.** The accumulated percentage of the feeding according to the number of fruit species in urban and rural bats (based on Additional file 2: Table S1).
