# Supplementary material for: Fruit bats adjust their foraging strategies to urban environments to diversify their diet
Source: BMC Biol. 2021 Jun 16;19:123. doi: 10.1186/s12915-021-01060-x (PMC8210355; doi:10.1186/s12915-021-01060-x)
Supplement: Supplementary file 7 — Additional File 7: Figure S6. Fruit bats select ripe fruit. A fruit bat feeding on a Ficus rubiginosa tree. Only red fruit (like the one in the bat’s mouth) was marked as ripe when estimating the amount of fruit. [file 12915_2021_1060_MOESM7_ESM.docx]

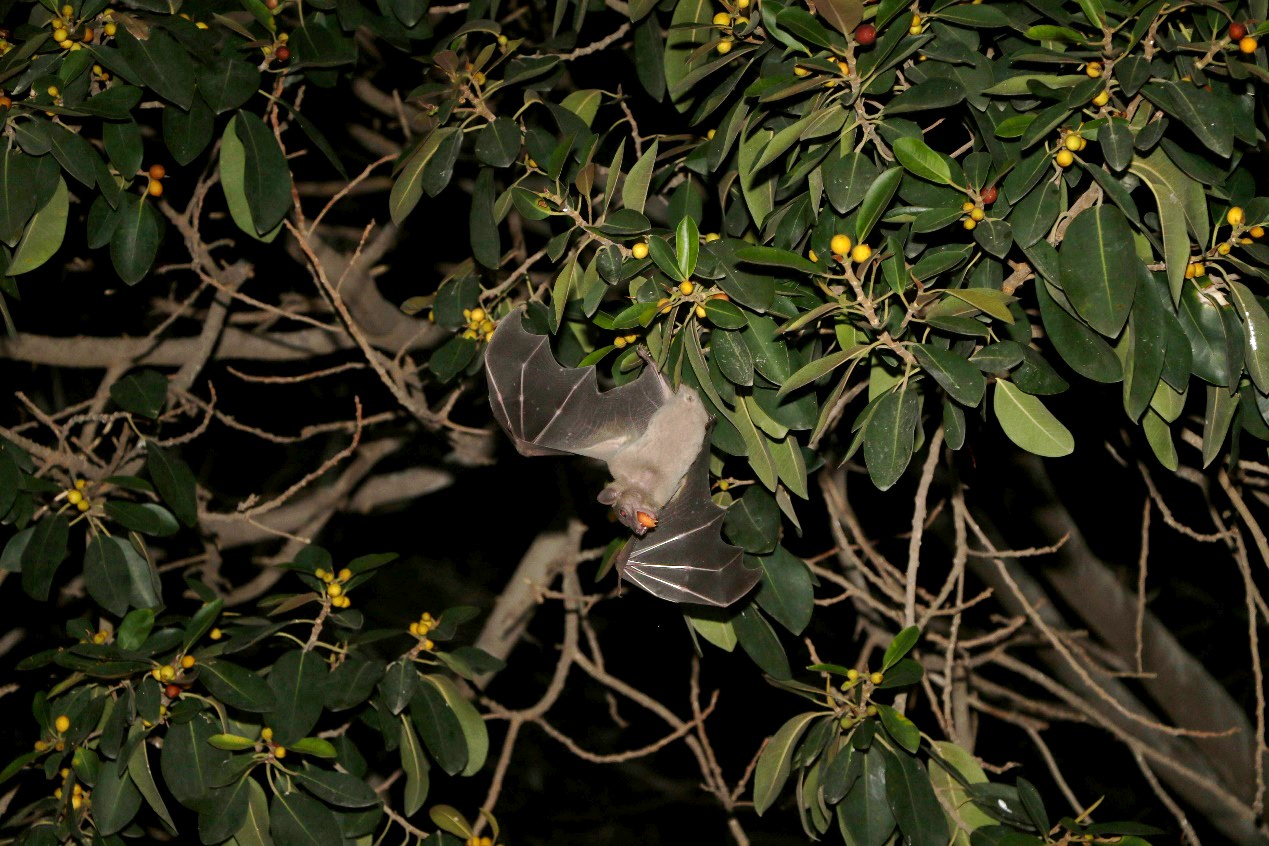


**Fig. 6. Fruit bats select ripe fruit.** A fruit bat feeding on a *Ficus rubiginosa* tree. Only red fruit (like the one in the bat’s mouth) was marked as ripe when estimating the amount of fruit.
